# Indexes for motor performance assessment in job integration/reintegration of people with neuromuscular disorders: A systematic review

**DOI:** 10.3389/fneur.2022.968818

**Published:** 2022-09-08

**Authors:** Giorgia Chini, Lorenzo Fiori, Antonella Tatarelli, Tiwana Varrecchia, Francesco Draicchio, Alberto Ranavolo

**Affiliations:** ^1^Department of Occupational and Environmental Medicine, Epidemiology and Hygiene, INAIL-Istituto Nazionale Assicurazione Infortuni sul Lavoro, Rome, Italy; ^2^Department of Physiology and Pharmacology and PhD Program in Behavioral Neuroscience, Sapienza University of Rome, Rome, Italy; ^3^Department of Human Neurosciences, Sapienza University of Rome, Rome, Italy

**Keywords:** indexes, job reintegration, neurological disorders, monitoring, performance, biomechanics, ergonomics

## Abstract

Individuals of working age affected by neuromuscular disorders frequently experience issues with their capacity to get employment, difficulty at work, and premature work interruption. Anyway, individuals with a disability could be able to return to work, thanks to targeted rehabilitation as well as ergonomic and training interventions. Biomechanical and physiological indexes are important for evaluating motor and muscle performance and determining the success of job integration initiatives. Therefore, it is necessary to determinate which indexes from the literature are the most appropriate to evaluate the effectiveness and efficiency of the return-to-work programs. To identify current and future valuable indexes, this study uses a systematic literature review methodology for selecting articles published from 2011 to March 30, 2021 from Scopus, Web of Science, and PubMed and for checking the eligibility and the potential bias risks. The most used indexes for motor performance assessment were identified, categorized, and analyzed. This review revealed a great potential for kinetic, kinematic, surface electromyography, postural, and other biomechanical and physiological indexes to be used for job integration/reintegration. Indeed, wearable miniaturized sensors, kinematic, kinetic, and sEMG-based indexes can be used to control collaborative robots, classify residual motor functions, and assess pre–post-rehabilitation and ergonomic therapies.

## Introduction

Individuals of working age with neuromuscular illnesses frequently struggle with employability, work challenges, and premature job stoppage (1–3). In any case, employment integration and reintegration have been shown to improve pathological people's overall quality of life (1, 4, 5). Indeed, increasing their working life should be an important element of neuromuscular disorder care in terms of psychological, social, and health wellness (6).

An increase in self-esteem and social wellness, as well as a reduction in workplace prejudice against disabled people, can be achieved by designing an adequate job accommodation (7–9), assistance and improving, among other things, the social environment, support from colleagues and supervisors, job expectations, and ergonomic interventions (10).

Furthermore, understanding of specific work-related difficulties, as well as focused rehabilitative, ergonomic, and training interventions, can enable individuals to return to work. Rehabilitation can play a constructive role by removing barriers to obtaining, retaining, or returning to work (11–18). This concept is supported by these people's contextual ability to maintain an effective motor strategy by adopting different compensatory behaviors during the disease, despite disease progression and motor decline (19–22). Neuropathies, multiple sclerosis, stroke, spastic paraplegia, cerebellar ataxia, dystonia, traumatic spine and brain lesions, and encephalitis are degenerative and acquired neurological diseases that can impact motor function throughout working age and severely limit workers' autonomy and efficiency (6, 23–27). Therefore, workers with neurological illnesses may have motor impairment in numerous motor domains, including hand function, balance, and locomotion, placing a significant burden on society in terms of lower job productivity and expense. Clinicians manage their patients' premature work interruptions (28, 29) by developing appropriate standard and new pharmacological, surgical, and rehabilitation treatments, such as robotic rehabilitation, virtual reality, and neuromodulation (30–34). Indeed, these treatments have the primary goal of restoring patients' motor performance, autonomy, and everyday life, allowing them to return to work and optimize their work capabilities.

Furthermore, novel ergonomic solutions, such as work task rehabilitation and workplace interventions, are being added to job accommodation plans (35–37). Indeed, the fourth industrial revolution has lately opened up new occupational scenarios in which crucial human–robot collaboration (HRC) technologies, such as collaborative robots and exoskeletons, aid workers in their workplaces (3).

When a worker affected by a neurological pathology with motor disorders is reintegrated at work, an exhaustive assessment of his/her residual motor function is of primary importance to design and/or optimally adapt his/her workplace. Therefore, biomechanical and physiological indexes are useful for monitoring motor and muscle performance and verifying the effectiveness of interventions for job integration/reintegration (3).

Furthermore, the efficiency of these ergonomic interventions should be verified and monitored throughout time (3). Kinematic, kinetic, and surface electromyography (sEMG) measurements are now widely used in research laboratories by movement scientists and could be used more and more in clinical practice by health operators, to define quantitatively the form and degree of motor dysfunction, assess the complicated interaction between the fundamental deficit and the adaptive and compensating mechanisms, categorize patients based on their specific neurological condition, and finally monitor pre–post-treatment (3).

As a result, it is important to determine which of the indexes available in the literature are the most suited for assessing the effectiveness and efficiency of return-to-work programs. This research employs a systematic literature review process to suggest present and future important indexes to achieve this purpose.

## Materials and methods

This study was performed using the systematic review method proposed by the Preferred Reporting Items for Systematic Reviews and Meta-Analysis (PRISMA) (38).

### Literature search strategy

This systematic review considered English articles published from 2011 to March 30, 2021, and the literature search was performed in a systematic manner using the following selected databases: Scopus, Web of Science, and PubMed. According to the database, the annual article production related to this research is starting to grow significantly from 2011, which is the starting year of the analyzed period.

There were four issues of interest in this systematic review (39): job reintegration, indexes, neurological, and quality.

For each issue identified according to the method proposed in the study mentioned in (39), the following keywords were identified as related to that topic and used for online database searching:

- **Job Reintegration**: “Job Integration,” “Job Reintegration,” “work Integration,” “work Reintegration,” “workplace,” “Return to work Rehabilitation,” “work ability”;
- **Indexes:** “kinematic index,” “kinetic index,” “force index,” “sEMG index,” “surface electromyography index,” “motor index”;
- **Neurological:** “Neurological motor disease,” “Neurological motor disorders,” “Neuromuscular motor disease,” “Neuromuscular motor disorders”;
- **Quality:** “performance,” “monitoring,” “ergonomics,” “quantitative,” “instrumental”.

A total of two, three, and four groups of keywords (one for each issue) were combined in the literature search (1,657 combinations).

We then entered each combination of one, two, three, or four keywords into each of the selected online databases (PubMed, Scopus, and Web of Science) to search for articles.

### Screening criteria

The literature search was performed by G.C., L.F., A.T., T.V., F.D., and A.R. The articles obtained were imported into Mendeley, and duplicates were removed. Our search was limited to peer-reviewed journal publications, reviews, chapters of books, and conference proceedings. The collected publications were then screened in three steps: (i) the titles were assessed for relevance; (ii) the abstracts were considered; and (iii) the complete text were downloaded when the information was deemed relevant.

### Inclusion and exclusion criteria of articles in the review

Studies were considered eligible if they were written in English, and they investigated subjects using biomechanical and physiological quantitative indexes. The common goal of these eligible studies was to perform a quantitative evaluation of programs/strategies to make patients sufficiently able to return to work. Excluded were narrative and systematic reviews or meta-analyses and purely clinical studies not aimed at evaluating job placement/reintegration. Furthermore, the studies with the following characteristics were also excluded:

- studies that do not consider indexes (biomechanical and physiological indexes) of motor performance for job integration/reintegration;
- studies on simulated data and not on people;
- studies with all or almost all participants of non-working age (>67 years, since the maximum range of retirement age in Italy is 67 years for most professions);
- studies on children/teenagers (<18 years, since in Italy, it is forbidden to work if you are younger than 18 years);
- studies on only work risks assessment.

A total of six reviewers (G.C., L.F., A.T., T.V., F.D., and A.R.) individually evaluated the eligibility for all articles by assessing titles and abstracts. Disagreements among reviewers were resolved by scheduling meetings to discuss and solve them.

In addition to searching databases with the aforementioned keywords, once the authors had identified articles for inclusion in the systematic review, they also examined the bibliography of the selected articles to check whether there were any additional articles that could be included in this systematic review.

### Data extraction

From the articles selected as eligible, the authors extracted the data that provided detailed information for each study, using the Population Intervention Comparison Outcome (PICO) framework as a guide when analyzing the eligible articles (40).

More in detail, the authors followed the following steps to extract the data from the selected articles:

- The authors looked for an existing extraction form or tool to help guide them and used existing systematic reviews on our topic to identify what information to collect if they are not sure what to do (41, 42).
- Train the review team on the extraction categories and what type of data would be expected.
- The authors performed a pilot extraction to ensure data extractors were recording similar data and revised the extraction form if needed.
- The authors discussed any discrepancies in extraction throughout the process.
- The review team documented any changes to the process or the form, kept track of the decisions the team made, and the reasoning behind them.

At the end of this procedure, the extracted information included the following:

- characteristics of the participants involved in the study: number of subjects (N), gender (F and M), age (years), height (H) in meters, weight (W) in kg, and/or body mass index (BMI) in kg/m^2^;
- measurement details: motor task, parameters/indexes names and acronyms if applicable, instrumentation used, and investigated body part;
- aims of the study;
- findings of the study.

### Assessment of bias

A bias represents a characteristic of a study that can introduce a systematic error in the magnitude or direction findings. The potential risk of bias was assessed independently by six authors (G.C., L.F., A.T., T.V., F.D., and A.R.) according to the Cochrane Handbook for Systematic Reviews of Interventions (43) and by using the tool ROBINS-I (44, 45), which was developed for the risk of bias assessment of non-randomized studies of interventions. The authors assessed the following risks of bias (44, 45):

- confounding (D1): occurs when one or more prognostic factors can also predict the baseline intervention;
- selection of participants into study (D2): even though the effects of the interventions are the same, there will be a connection between interventions and outcomes when exclusion of some eligible participants, initial follow-up times for some participants, or certain outcome events are connected to both the intervention and the outcome;
- classification of interventions (D3): by misclassification of intervention status;
- deviation from the intended study (D4): when there are consistent discrepancies between experimental intervention and comparison groups, which represent a deviation from the intended intervention;
- missing data (D5): when later follow-up or information is missing for individuals initially included and followed;
- measurement of outcomes (D6): introduced by errors in measurement of outcome data;
- selection of the reported result (D7): selective reporting of results in a way that depends on the findings.

## Results

### Study selection

**Figure 1:**
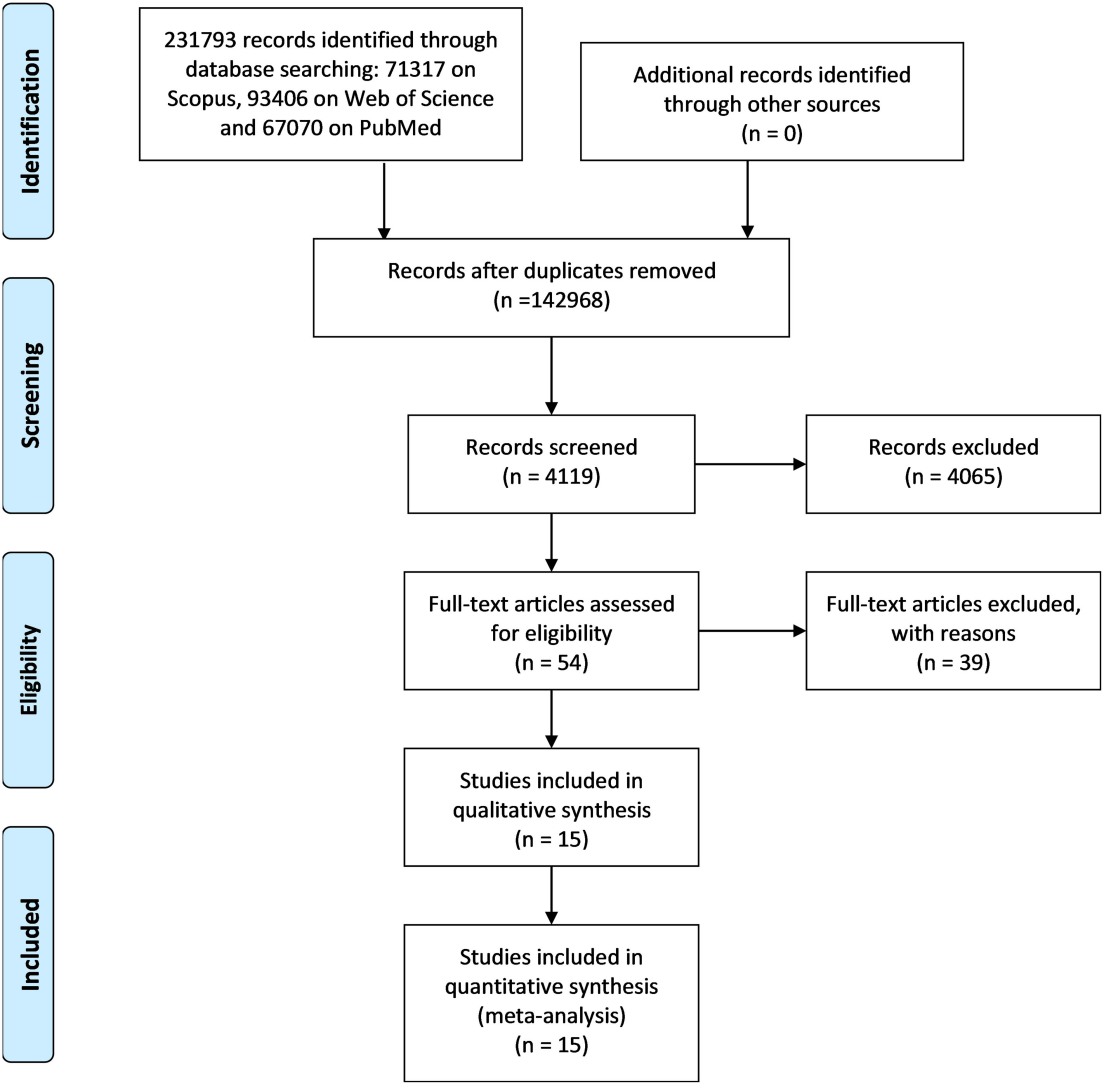
PRISMA flowchart related to the steps of a systematic review provided by the journal Frontiers in Neurology.

The study selection process started from the results of the literature database search that yielded 231,793 records, as shown in Figure 1. In particular, 71,317 were found on Scopus, 93,406 on Web of Science, and 67,070 PubMed. After removing the duplicates, the articles were 142,968. These articles were screened by deleting articles on the basis of not connected words (e.g., animal, astronomy, and human resources) and journals (e.g., International Journal of Molecular Science), obtaining 4,119 articles.

These articles were screened based on their title, obtaining 1,187 articles. From this group, abstracts were read, and 1,133 were excluded by the screening criteria. Consequently, 54 full-text articles were assessed for eligibility. Finally, after having removed 39 articles by the eligibility criteria, a total of 15 articles were included in this systematic review.

### Characteristics of the studies

Table 1 shows an overview of the main characteristics of the 15 considered studies (46–60) following the PICO model (40) and highlighting the biomechanical and physiological indexes used. All the articles that met the eligibility criteria are very recent: five were published in 2020, one in 2019, six between 2013 and 2016, and three, the oldest, in 2012.

**Table 1:** Descriptive analysis of the studies considered in the review according to the PICO method.

**References**	**Participants involved in the study**	**Measurement details**				**Aims**	**Findings**
		**Motor task**	**Parameters/indexes name (acronym)**	**Instrumentation**	**Body part**		
Monticone et al. (46)	*N* = 20: • 10 LBP (7 F, 3 M; Age = 58.9 ± 16.4 y; BMI = 27.4 ± 4.9 kg/m^2^); • 10 HS (4 F, 6 M; Age = 56.6 ± 14.4 y; BMI = 25.2 ± 3.1 kg/m^2^).	Spinal stabilizing exercises in addition to usual-care rehabilitation (passive mobilisatio, stretching, and postural control); • Individual cognitive–behavioral training.	**Gait parameters**: velocity, cadence, step length, step time, and single support time of both sides.	GAITRite-Walkway System (CIR System Inc., Clifton, NJ).	-	To evaluate the effect of a multidisciplinary rehabilitation program on disability, kinesiophobia, catastrophizing, pain, quality of life and gait disturbances in patients with chronic LBP.	The findings indicate that the treatment was beneficial in terms of gait cadence, as well as the positive impact of cognitive–behavioral therapy on non-spinal motor tasks, which improved health and favored a return to work and usual activities.
Lee et al. (47)	*N* = 35 HS: • 18 (5 F, 13 M; Age = 21.7 ± 2.3 y) participated in the in-laboratory experiments; • 18 (4 F, 14 M; Age = 23.4 ± 4.2 y) in the free-living experiments; one subject participated in both.	**• Laboratory experiments:** Walking; Buttoning a shirt Bilateral; Tying shoelaces; Typing on a keyboard; Folding a towel; Cutting putty dough with a fork and a knife; Opening a screw-top jar; Taking the cap off of a bottle and drinking; Flipping pages of a magazine. • **Free-living experiments:** Normal daily routines.	The ratio of limb use; • Limb-use intensity (i.e., acceleration magnitude).	Miniaturized sensor (Arcus, ArcSecond Inc., USA) consisted of a three-axis accelerometer, a local memory for data storage, a 170 mAh battery, and an ultra-low-power 32-bit microprocessor in a waterproof enclosure.	Hands.	To investigate the use of finger-worn accelerometers to monitor gross arm and fine hand movement; to examine the validity of the proposed approach by collecting and analyzing data from neurologically intact individuals in a laboratory and a free-living environment as a preliminary step toward developing a system suitable to monitor stroke survivors in the home and community setting; to describe a comprehensive approach integrating both a clinical- and functional status-based pathology and an adapted rehabilitation prescription.	The results establish the validity of the proposed measure of real-world upper-limb function derived using data collected by means of finger-worn accelerometers.
Richmond et al. (48)	*N* = 56: • 29 HS (21 F, 8 M; Age = 47 ± 15 y; *H* = 1.69 ± 0.08 m; *W* = 72.4 ± 14.2 kg; BMI = 25.3 ± 4.0 kg/m^2^); • 27 MS (20 F, 7 M; Age = 48 ± 12 y; *H* = 1.66 ± 0.08 m; *W* = 68.6 ± 9.2 kg; BMI = 24.9 ± 3.8 kg/m^2^).	Walking task.	Phase coordination index (PCI).	Six tri-axial Opal™ body-worn inertial monitoring units (IMUs).	Sternum, lower back (L4/L5 region), wrists and feet.	To identify the temporal actions underlying bilateral coordination in people with MS and how bilateral coordination is affected by gait speed augmentation in these individuals.	People with MS exhibited poorer left-right coordinated stepping patterns during gait compared to neurotypical peers across walking conditions. This assessment highlights Phase Coordination Index as a potential target for future rehabilitative interventions for subjects with MS and individualized rehabilitation strategies aimed at improving the health span and overall quality of life for subjects with MS.
Cimarras-Otal et al., (49)	*N* = 18: • 10 LBP (2 F, 8 M; Age = 42.25 ± 7.28 y; *H* = 1.69 ± 0.05 m; *W* = 72.75 ± 15.79 kg; BMI = 25.12 ± 4.69 kg/m^2^); • 8 HS (4 F, 4 M; Age = 42.20 ± 5.59 y; *H* = 1.68 ± 0.09 m; *W* = 68.27 ± 12.80 kg; BMI = 23.80 ± 2.34 kg/m^2^).	Flexion-lumbar extension.	• Angle and flexion; • Bending speed; • Root mean square (RMS) of EMG signal; • Angle, bending speed, and flexion-extension ratio (FER).	SMART-DX (BTS Bioengineering, Italy): BTS FREEEMG 300 electromyographic probes; Six BTS Bioengineering—SDX-C2 3D; Two video cameras BTS VISTA.	Trunk.	To investigate whether an exercise program adapted to the characteristics of the workplace is a useful supplement to general exercise recommendations in assembly line workers with chronic LBP.	Results demonstrated that the implementation of a physical exercise program adapted to the characteristics of the workplace, for workers with chronic LBP, could be an effective treatment to reduce the interference of pain and to improve the functionality of the lumbar spine.
Schaefer et al. (50)	*N* = 28: • 16 subjects with SS (7 F, 9 M; Age = 58 ± 11 y); • 12 HS (6 F, 6 M; Age = 53 ± 16 y).	Reach-to-grasp.	• **Reaching performance**: reach path ratio, peak reach velocity, reach time, contact velocity; • **Grasping performance**: peak aperture, peak grip force.	Electromagnetic tracking system with nine sensors (The Motion Monitor, Innovative Sports Training, Chicago, IL).	Midsternum; upper arm; forearm; hand; fingernail of each digit.	To determine whether performance of a functional reach-to-grasp movement in people with poststroke hemiparesis is influenced by grip type and/or task goal; to directly test how stroke might alter patterns of performance when moving with multiple grip types and task goals.	Results suggest that even though the ability to move one's arm and hand is often impaired after stroke, reaching and grasping performance can still be modified based on how and why an object will be grasped. Information about how different movement contexts influence performance poststroke may assist therapists in planning how and what to practice during task specific upper extremity training.
Correia et al. (51)	13 SCI at level C4–C7 (Age = 54.54 ± 16.23 y).	Grasping.	• Activities of daily living using the Jebsen Taylor Hand; • Active range of motion of the fingers; • Grasp strength for power; • Pinch grasps.	SOFT ROBOTIC GLOVE, Goniometer, pressure sensor mat.	Hand.	To evaluate the performance of the optimized soft robotic glove in restoring activities of daily living for individuals with tetraplegia resulting from SCI.	Results demonstrated the effectiveness of a fabric based soft robotic glove to improve independent performance of activities of daily living in individuals with hand paralysis resulting from SCI.
Kim and Martin (52)	*N* = 29: • 10 HS (7 M, 3 F; Age = 28.0 ± 11.3 y; *W* = 81.3 ± 20.3 kg); • 10 SCI (10 M; Age = 39.0 ± 13.7 y; *W* = 75.2 ± 17.4 kg). • 9 LBP (5M, 4F; Age = 47.8 ± 11.6 y; *W* = 84.0 ± 29.2 kg).	Manually moving a hand-held box from an initial position to one of four target shelves.	Precedence Index (PI).	-	Upper body segments.	To characterize the temporal coordination between the torso and hands in SCI and LBP individuals.	Results demonstrated that hands and torso movements show adapted patterns of coordination in the population with injury. Altogether, it is suggested that patterns of temporal coordination, can be effectively used to assess the gravity of injury, progress of rehabilitation and work capacity measurements.
Bruce-Low et al. (53)	*N* = 72 LBP (42 M, 30 F; Age = 45.5 ± 14.1 y).	• Maximal lumbar isometric strength; • modified-modified Schober's flexion test; • completion of the Oswestry disability index (ODI); • the visual analog scale (VAS).	Maximal Strength; • Range of Motion (ROM); • Scober's flexion.	Lumbar extension machine (MedX, Ocala, FL).	Lumbar part of the spine.	To examine whether the second weekly dynamic training session is actually beneficial in increasing isometric strength, range of motion (ROM) and decreasing perceived pain in subjects with chronic LBP.	Results suggest that in the rehabilitation of workers suffering from chronic lower back pain, resistance training of the lumbar muscles improves isometric strength and ROM.
Lebde et al. (54)	*N* = 720 HS (364M, 356F; Age = 52.3 ± 20.9 y; *W* = 71.4 ± 14.0 kg; *H* = 1.69 ± 0.1 m; BMI = 24.8 ± 3.8 kg/m^2^).	Isometric muscle strength of 13 muscle groups; • flexibility of six joints; • 11 functional measures classified as gross motor, fine motor or balance tasks.	• **Isometric muscle strength (N):** Shoulder internal/external rotation, Elbow flexion/extension, Grip; Hip abduction, Hip internal/external rotation, Knee flexion/extension, Ankle dorsi/plantarflexion, Toe flexion; • **Joint flexibility:** Neck flexion/extension, Shoulder internal/external rotation, Elbow flexion/extension, Hip flexion, Hip internal/external rotation, Knee flexion/extension, Ankle dorsi/plantarflexion, walk distance, gait velocity.	Fixed dynamometry (CSMi; HUMAC NORM, Stoughton, Massachusetts, USA); Hand-held dynamometry (Citec dynamometer CT 3001; CIT Technics, Groningen, the Netherlands); A universal goniometer (Baseline, Fabrication Enterprises, White Plains, New York, USA) or digital inclinometer (ankle dorsiflexion lunge test).	Full body.	To generate an age-stratified dataset of normative reference values for work ability in a healthy adult Australian population using the Work Ability Score (WAS) and investigate the association of physical performance factors.	Results identified physical factors associated with work ability that can potentially be targeted to maintain longevity in work. Physical tests may assist in the development of objective job-specific screening tools to assess work ability, supplementing subjective evaluation.
Taylor-Piliae et al. (55)	*N* = 20: • 10 HS (2M, 8F; Age = 74.0 ± 7.0 y); • 10 SS (3M, 7F; Age = 70.0 ± 8.0 y).	Daily activities.	• Trunk tilt (°); • Type of the participant's postural transitions (e.g., sit-to-stand); • Duration of the participant's postural transitions; • Duration of the participant's locomotion; • Characterization of the participant's locomotion (gait speed and number of steps); • Type of the participant's postures (walking, sitting, standing, lying).	Kinematic motion sensor (PAMSys, Biosensics LLC, MA, USA)	Trunk.	To determine the feasibility of using a kinematic motion sensor to objectively monitor fall risk and gait in naturalistic environments in community-dwelling stroke survivors.	Results highlight the utility of using objective kinematic motion sensors to monitor fall risk and gait in community-welling stroke survivors—so that strategies can be implemented early on, to reduce the risk of falling in this vulnerable population. As sensor algorithms become increasingly more predictive with less obtrusive applications, for home and community settings.
Brooks et al. (56)	*N* = 64 LBP: • 32 in Group1 (12 M, 20 F; Age = 36.2 ± 8.2 y; *H* = 171 ± 8.0 cm; *W* = 80.0 ± 13.8 kg); • 32 in Group2 (12 M, 20 F; Age = (Missing Data) ± 6.3 y; *H* = 171 ± 9.0 cm; *W* = 85.5 ± 17.8 kg).	• Specific Exercise Group (SEG); • general Exercise Group (GEG).	The onset time.	Electromyography ML138 Bio Amp (common mode rejection ratio >85 dB at 50 Hz, input impedance 200 M Ω) with 16-bit analog-to-digital conversion, sampled at 2000 Hz (ADI instruments, Analog Digital Instruments, Sydney, Australia).	Trunk.	To measure self-rated disability, pain, and the onsets of various trunk muscles in response to a rapid shoulder movement as a measure of anticipatory postural adjustments (APAs), before and after 8 weeks of specific trunk or general exercise in patients with LBP. To verify that that selfrated disability and pain scores would decrease after specific trunk exercise and APAs, whether delayed or not at baseline, would change only after specific trunk exercise	Results show similar between-group changes in trunk muscle onsets were observed. The motor control adaptation seems to reflect a strategy of improved coordination between the trunk muscles with the unilateral shoulder movement. Trunk muscle onsets during rapid limb movement do not seem to be a valid mechanism of action for specific trunk exercise rehabilitation programs
Shin and Sosnoff (57)	*N* = 36: • 18 HS (10 M, 8 F; Age = 22.14 ± 3.07 y; Sitting *H* = 84.95 ± 4.65 cm; *W* = 63.03 ± 8.15 kg); • 7 High SCI (5 M, 2 F; Age = 23.27 ± 3.67 y; Sitting *H* = 78.56 ± 9.57 cm; *W* = 62.87 ± 13.35 kg); • 11 Low SCI (5 M, 6 F; Age = 21.36 ± 2.29 cm; Sitting *H* = 86.13 ± 10.95 cm; *W* = 62.88 ± 9.79 kg).	• Functional reach test; • leaned forward, backward, side to side, and diagonally by pivoting at the hip joints to trace a circle while leaning as far as possible without losing balance for 1 min; • sitting still for 30	• Center of pressure (CoP); • Root mean square (RMS); • Median velocity; • Virtual time to contact (VTC); • Instability index.	Force platform; AMTI, Inc., 176 Waltham St, Watertown, MA 02472-4800.	Upper body.	To investigate seated postural control in persons with SCI compared with age-matched controls.	Results suggest that VTC analysis is appropriate to investigate seated postural control. It is proposed that including VTC of seated postural control as an outcome measure will provide novel information concerning the effectiveness of various rehabilitation approaches and/or technologies aimed at improving seated postural control in persons with SCI.
Moreside et al. (58)	*N* = 81 • 30 LBP (14 M, 16 F; Age = 40.7 ± 12 y; *H* = 169.9 ± 9 cm; *W* =7 7.6 ± 20 kg; BMI = 26.6 ± 6 kg/m^2^); • 51 HS (24 M, 27 F; Age = 31.5 ± 8 y; *H* = 171.1 ± 9 cm; *W* = 71.5 ± 15 kg; BMI = 24.3 ± 4 kg/m^2^).	Trunk stability test.	EMG principal component score.	Surface electrodes (Meditrace silver/silver chloride electrodes); 3 AMT-8 EMG systems; An electromagnetic Flock of Birds Motion Capture system.	Trunk.	To compare temporal activation patterns from 24 abdominal and lumbar muscles between healthy subjects and those who reported recovery from recent low back injury.	Results demonstrated that despite perceived readiness to return to work and low pain scores, muscle activation patterns remained altered in this low back injury group, including reduced synergistic coactivation and increased overall amplitudes as well as greater relative amplitude differences during specific phases of the movement. Electromyographic measures provide objective information to help guide therapy and may assist with determining the level of healing and return-to-work readiness after a low back injury.
Rowley et al. (59)	*N* = 38: • 19 LBP (7 M, 12 F; Age = 23.5 ± 2.8 y; *H* = 170.4 ± 8.4 cm; *W* = 68.7 ± 10.3 kg; BMI = 23.6 ± 2.47 kg/m^2^); • 19 HS (2 M, 12 F; Age = 23.9 ± 3.3 y; *H* = 169.1 ± 10.4 cm; *W* = 67.1 ± 10.8 kg; BMI = 23.3 ± 1.8 kg/m^2^).	The Balance-Dexterity Task protocol.	Mean muscle activation.	Surface EMG (Noraxon Wireless EMG; Scottsdale, AZ; 3,000 Hz); Advanced Medical Technology Inc. force plates (Watertown, MA; 3,000 Hz).	Trunk and hip.	To examine the association between hip and trunk muscle activity during dynamically perturbed single-limb balance using the Balance-Dexterity Task in persons with and without LBP.	Results demonstrated that there were no between-group differences in activation amplitude for any muscle groups tested. Back-healthy control participants increased hip and trunk muscle activation amplitudes in response to the added instability of the spring in a coordinated way, while those in remission from LBP did not. Instead, hip muscle activation and task performance were associated in those with LBP. These findings suggest persons with LBP preferentially, and potentially excessively, utilize hip musculature during challenging dynamic balance tasks. This represents an extrapolation of previous findings where persons with symptomatic LBP had greater hip muscle activity than controls, and this may help explain the dissociated trunk motion observed in those in remission from LBP during the Balance-Dexterity Task.
Hubley-Kozey et al. (60)	*N* = 70: • 35 LBP (Age = 39.6 ± 12 y; *H* = 170.3 ± 9 cm; *W* = 79.3 ± 21 kg; BMI = 27.2 ± 6 kg/m^2^); • 35 HS (Age = 35.5 ± 10 y; *H* = 171.7 ± 8 cm; *W* = 76.7 ± 15 kg; BMI = 25.9 ± 4 kg/m^2^).	Highly controlled right-to-left transfer task.	• EMG ensemble average waveforms; • PCA model.	Surface electrodes (Ag/AgCl, 10 mm circular electrodes; Meditrace, Graphics Control Canada Ltd.); Electromagnetic Flock of BirdsTM (FOB) Motion Capture system (Ascension Technology Inc., Burlington, Vermont).	Low back.	To determine if amplitude and temporal characteristics of trunk neuromuscular patterns differ during a dynamic functional task in a group of participants with recent (within 12 weeks) low back injury, but deemed ready to resume normal activities, when compared to those with no similar history of injury (ASYM).	Results demonstrated that despite the perception of readiness to return to work and low pain scores, the temporal and amplitude muscle activation patterns were altered in this low back injury group indicating that differences exist compared to a non-low back injured group. The differences are not just relative amplitude differences among muscles but include differences in the temporal response to the flexion moment.

F, female; M, male; H, height; W, weight; BMI, body mass index; LBP, subjects with low back pain; SS, subjects who have survived stroke; MS, subjects with multiple sclerosis; SCI, subjects with spinal cord injury; HS, healthy subjects.

A total of 1,300 subjects were recruited in the included studies, with 618 males (M) and 612 females (F), and only in one study (60), the gender was not specified. The subjects' mean age varied from <21.36 (57) to 74 (55) years.

A total of eight studies dealt with subjects with low back pain (LBP) (46, 49, 52, 53, 56, 58–60), two studies considered subjects who have survived stroke (SS) (50, 55), one study considered subjects with multiple sclerosis (MS) (48), three subjects with spinal cord injury (SCI) (51, 52, 57), and finally, two addressed healthy subjects (HS) (47, 54).

All studies were carried out in the laboratory (46–60), and three of them also in real-life environments (47, 52, 55).

The following tasks were analyzed (see Table 1):

- lifting task, one study (60);
- rehabilitation exercises, three studies (46, 53, 56);
- daily activities, two studies (47, 55);
- walking task, two studies (47, 48);
- balance, three studies (54, 57, 59);
- reaching and grasping activities, three studies (50, 51, 57);
- typical working activities, one study (52);
- lumbar flexion–extension, one study (49);
- physical performance task, one study (54);
- trunk stability test, one study (58);
- gross arm and fine hand movements, one study (47);
- sitting test, one study (57);
- dexterity task, one study (59).

In total, 41 different kinematic (46–55, 57, 60), 12 kinetic (51, 53, 54, 57), 5 sEMG (46, 56, 59, 60), 3 postural (55, 57), and 4 other indexes (47, 49, 50) were investigated (see Tables 1, 2).

**Table 2:** Description of all the outcome parameters from the eligible studies.

**Outcome type**	**Outcome measure**	**Unit**	**Description and/or calculation**	**Neuromuscolar disorder**	**References**
Kinematic	Velocity	(m/s)	Speed adopted by the subject to walk.	LBP/MS/HS/SS	(46, 48, 54, 55)
	Number of steps and cadence^#^	(a.u.)	Number of steps performed during the test or in 1 min.	SS/LBP	(46, 55)
	Gait duration	(% of total activity)	Percentage of total activity dedicated to walking.	SS	
	Step time^#^	(s)	The time between the point of initial contact of one foot and the point of initial contact of the opposite foot.	LBP	
	Single and double support duration	(s), (%GCT: Gait cycle time)	The time or gait cycle percentage during which just one foot or both feet are in touch with the ground.	LBP/MS	(46, 48)
	Stance duration	(%GCT: Gait cycle time)	The gait cycle percentage during which the foot is in contact with the ground.	MS	(46, 48)
	Swing duration	(%GCT: Gait cycle time)	The gait cycle percentage during which the foot is not in contact with the ground.	MS	
	Stride and step length	(m)	The distance between successive points of initial contact of the same foot or between the points of initial contact of one foot and the opposite foot.	MS/LBP	
	Phase coordination index	(%)	Bilateral limb coordination calculated by modeling the gait cycle as 360 degrees with a step equating a phase (ϕ) within the cycle: *PCI* = φ_*CV*_+ *P*_φ_*ABS*__[% ] where ϕ_ABS and ϕ_CV represent the accuracy and consistency of phase generation, respectively	MS	
	Limb-use intensity	(m/s^2^)	Limb acceleration magnitude calculated as follows: $|a_{l}|=\;\sqrt{a_{l,x}^{2}+a_{l,y}^{2}+a_{l,z}^{2}}-g\;$; $|a_{r}|=\;\sqrt{a_{r,x}^{2}+a_{r,y}^{2}+a_{r,z}^{2}}-g$ where *a*_*r*_[*t*] and *a*_*l*_[*t*] are the accelerations of the right and left limbs respectively.	HS	(47)
	Flexion angle	(°)	Measurement of maximum lumbar flexion recorded through a motion capture system	LBP	(49)
	Bending/flexion speed	(°/s)	Speed at which forward bending is performed measured with a motion analysis system.	LBP	
	Peak reach velocity	(mm/s)	Maximum three-dimensional resultant velocity of the hand during the reach.	SS	(50)
	Reach time	(ms)	Duration from reach start to reach end.	SS	
	Contact velocity	(mm/s)	Three-dimensional resultant velocity of the hand at reach end.	SS	
	Peak aperture	(mm)	Maximum three-dimensional distance between the thumbnail and the index fingernail during the reaching phase.	SS	
	Peak grip force	(grams)	Maximum grip force of the object during the hold task or the lift task.	SS	
	Fingers range of motion	(°)	Active range of motion (ROM) of the fingers measured with a goniometer for the metacarpophalangeal (MCP) and proximal interphalangeal (PIP) joints of the index finger, and for the MCP joint of the thumb.	SCI	(51)
	Torso travel distance	(cm)	The distance traveled by the torso from the initial position (*t* = 0) to reaching the target (*t* = T), calculated as follow: $D\left(T\right)=\int_{0}^{T}||\overset{∙}{\text{p}}\left(t\right)||dt\;$ Where $\left||\overset{∙}{\text{p}}\left(t\right)|\right|$ is the magnitude of the C7/T1 landmark's instantaneous velocity vector.	SCI	(52)
	Hand travel distance	(cm)	The distance traveled by the Hand from the initial position (*t* = 0) to reaching the target (*t* = T), calculated as follow: $D\left(T\right)=\int_{0}^{T}||\overset{∙}{\text{p}}\left(t\right)||dt\;$ Where $\left||\overset{∙}{\text{p}}\left(t\right)|\right|$ is the magnitude of the instantaneous velocity vector of the right hand grip.	SCI	
	Movement duration	(s)	The difference between the start and end movement times.	SCI	
	Hand peak velocity	(cm/s)	Maximum speed achieved by the hand.	SCI	
	Torso peak velocity	(cm/s)	Maximum speed achieved by the Torso.	SCI	
	Time at hand peak velocity	(a.u.)	Time instant (normalized to movement time) at which the maximum velocity is achieved by the hand.	SCI	
	Time at torso peak velocity	(a.u.)	Time instant (normalized to movement time) at which the maximum velocity is achieved by the Torso.	SCI	
	Shoulder-to-hand distance at hand peak velocity	(cm)	The distance between the shoulder (acromion process) and the hand (middle point of the dorsal surface) at the maximum hand speed.	SCI/LBP	
	Precedence index (PI)		Index expressing the coordination of movement between torso and hand. PI = 0 when the hand and torso move in synchrony. PI > 0 indicating that hand movement precedes torso movement. In contrast, when the torso precedes the hand, PI is <0. calculated as follow: $PI=\frac{1}{T_{MT}}\int_{0}^{T_{MT}}\left[N_{hand}\left(t\right)-N_{Torso}\left(t\right)\right]dt\;$ where *N*_*hand*_(*t*) and *N*_*Torso*_(*t*) indicate the normalized travel distance of the hand and torso at time t, respectively, and *T_*MT*_* denotes the total movement time.	SCI/LBP	
	Lumbar ROM	(°)	Lumbar movement range calculated with the goniometer within the MedX lumbar extension machine.	LBP	(53)
	Schober's flexion	(cm)	ROM of the lumbar spine. In order to undertake the modified-modified Schober's test pen marks were made at each of the posterior superior iliac spines (PSIS). Another mark was made at the midline of the lumbar spines horizontal to the PSIS and a final mark was then made 15 cm above this mark. Whilst holding a tape measure close to the participant's skin, he or she bent over as though to touch the toes whilst a reading was obtained to ascertain any change in the original 15 cm measure.	LBP	
	Flexion/extension of neck, hip and knee	(°)	Joint flexibility of the neck, hip and knee in terms of range of motion, measured using a universal goniometer or digital inclinometer	HS	(53)
	Shoulder and hip internal/external rotation	(°)	Joint flexibility of the shoulder and hip in terms of range of motion, measured using a universal goniometer or digital inclinometer	HS	
	Ankle plantar/dorsiflexion	(°)	Joint flexibility of the Ankle in terms of range of motion, measured using a universal goniometer or digital inclinometer.	HS	
	Walk distance	(m)	Distance walked during the 6-min walking test.	HS	
	Functional boundary	(mm^2^)	Stability area drawn during movements, performed in a sitting position, forward, backward, diagonally and side to side without losing balance, calculated using a direct least square fitting method.	SCI	(57)
	Virtual time to contact (VTC)	(s)	Parameter that specifies the spatiotemporal proximity of the CoP to the postural stability boundary taking into account acceleration, velocity, and position of the COP trajectory.	SCI	
	Maximum angular displacement	(°)	Maximum angular displacement of hands in the three planes of space measured with an electromagnetic Flock of BirdsTM (FOB) Motion Capture system.	LBP	(60)
Kinetic	Palmar maximum grasp strength	(N)	Maximum force expressed in the palmar grasp.	SCI	(51)
	Pinch maximum grasp strength	(N)	Maximum force expressed in the pinch grasp.	SCI	
	Maximal voluntary isometric torque	(N*m)	The extension torque expressed by the trunk, in isometric condition. Calculated at intervals of 12° from 0° to 72° of lumbar flexion with a 10 s rest between each joint angle.	LBP	(53)
	Muscle strength shoulder internal/external rotators	(N)	Physical performance of the shoulder muscles measured using hand-held dynamometry.	HS	(54)
	Muscle strength elbow flexors/extensor	(N)	Physical performance of the elbow muscles measured using hand-held dynamometry.	HS	
	Muscle strength hip abductors	(N)	Physical performance of the hip muscles measured using hand-held dynamometry.	HS	
	Muscle strength hip internal/external rotatators	(N)	Physical performance of the hip muscles measured using hand-held dynamometry.	HS	
	Muscle strength Knee flexor/extensor	(N*m)	Physical performance of the knee muscles measured using fixed dynamometry.	HS	
	Muscle strength Ankle plantar-flexors/dorsiflexors	(N)	Physical performance of the Ankle muscles measured using hand-held dynamometry.	HS	
	Muscle strength Toe flexor	(N)	Physical performance of the Toe muscles measured using the Paper Grip Test and a composite score out of six was summed based on the number of successful trials of Paper Grip Test-1 (hallux strength) and Paper Grip Test-2 (lesser toes strength).	HS	
	Center of pressure (CoP) velocity	(mm/s)	Center of pressure speed.	SCI	(57)
	CoP Root mean square (RMS)	(mm)	The mean square error of the CoP's trajectory described during task execution between the subject and the seat equipped with a force platform.	SCI	
Electromyography	Muscles onset	(ms)	The onset of the abdominal and lumbar muscles during rapid right arm shoulder flexion was measured using surface electromyography.	LBP	(56)
	Latency time	(ms)	The time between the onset of each trunk muscle and the anterior deltoid.	LBP	
	Principal component score	(a.u.)	Abdominals and back extensors muscles score, calculated with the principal component analysis model, that provide a weighting factor for the contribution of the Principal Component to the measured EMG waveform.	LBP	(58, 60)
	Mean muscle activation	(%)	Average activation of hip and trunk muscles during the Balance-Dexterity Task protocol measured with surface and fine-wire electromyography. Muscle activations were reported as a percent of activation during the stable block condition and thus represent additional muscle activation utilized in response to instability of the spring	LBP	(59)
	EMG ensemble average waveforms	(%MVIC)	Average waveforms for the right sided abdominal and back extensor muscles recorded with surface electrodes.	LBP	(60)
Postural	Postural transition duration	[s]	Postural transition duration are identified by measuring the pattern recognition of the trunk tilt with kinematic motion sensor PAMSys.	SS	(55)
	Aborted postural transition attempts	(Number/day)	Unsuccessful attempts rising from a chair quantified by kinematic motion sensor PAMSys.	SS	
	Instability index	(a.u.)	The ratio of the area defined by the COP's trajectory described during the task to the functional boundary; high index values indicate poor postural stability.	SCI	(57)
Other	Mean of magnitude ratio of activity intensity	(a.u.)	Mean of magnitude ratio of the acceleration of one limb in comparison to the other during daily activities and calculated as the mean of this quantity: *r*[*t*] = *ln*(|*a*_*r*_[*t*]|)−*ln*(|*a*_*l*_[*t*]| ) where *a*_*r*_[*t*] and *a*_*l*_[*t*] are the accelerations of the right and left limbs, respectively.	HS	(47)
	Upper-limb performance	(a.u.)	A measure of how much one limb is used in comparison to the other during activities of daily living calculated as follow: $M=\frac{\left|t\in \left\{r\left[t\right]>\delta ,|a_{r}\left[t\right]|>\beta \right\}\right|}{T}-\frac{\left|t\in \left\{r\left[t\right]<-\delta ,|a_{l}\left[t\right]|>\beta \right\}\right|}{T}\;$ where *r*[*t*] , *a*_*r*_[*t*] and *a*_*l*_[*t*] are the magnitude ratio of activity intensity and accelerations of the right and left limbs, respectively. T is the total monitoring duration in seconds, β represents a parameter identifying upper limb activities (epochs) and δ is a threshold.	HS	
	Flexion-extension ratio (F/R ratio)	(a.u)	Ratio between the maximum EMG value during flexion and the minimum resting EMG.	LBP	(49)
	Reach path ratio	(a.u.)	Total distance traveled by the wrist sensor divided by the length of a straight-line path from the reach's starting point to ending point.	SS	(50)

LBP, subjects with low back pain; SS, subjects who have survived stroke; MS, subjects with multiple sclerosis; SCI, subjects with spinal cord injury; HS, healthy subjects.

# Refer to similar information.

### Motor performance assessment in return-to-work programs: The outcome parameters

All the outcome parameters of interest are listed and described in Table 2.

#### Kinematic parameters

In 13 studies, five on LBP (46, 49, 52, 53, 60), two on SS (50, 55), one on MS (48), three on SCI (51, 52, 57), and two on HS (47, 54), 31 kinematic indexes were reported as useful for motor performance assessment (Table 2). More in detail, with regard to the gait: velocity (46, 48, 54), number of steps (55), gait duration (55), cadence (46), step length (46), step time (46), single support time (46), stride length (48), and phase coordination index (48). With regard to other motor tasks, different from gait: limb-use intensity (47), flexion angle (49), bending/flexion speed (49), peak reach velocity (50), reach time (50), contact velocity (50), peak aperture (50), peak grip force (50), fingers range of motion (51), movement duration (52), hand peak velocity (52), torso peak velocity (52), time at torso peak velocity (52), shoulder-to-hand distance at hand peak velocity (52), precedence index (52), lumbar ROM (53), Schober's flexion (53), neck flexion/extension (54), shoulder external rotation (54), hip flexion, internal/external rotation (54), knee flexion/extension (54), virtual time to contact (VTC) (57), and maximum angular displacement (60).

The following kinematic parameters were found to be less significant for characterizing the motor performance of analyzed subjects. Regarding the gait: velocity (55), single support time (48), stance (48), swing (48), double support time (48), and walk distance (54). In other tasks: bending/flexion speed (49), torso travel distance (52), hand travel distance (52), time at hand peak velocity (52), shoulder internal rotation (54), ankle plantar/dorsi flexion (54), and functional boundary (57).

#### Kinetic parameters

In three studies, one on LBP (53), one on SCI (51), one on HS (54), and five kinetic indexes have been identified as useful for the assessment of motor functions in tasks not including gait: palmar maximum grasp strength (51), pinch maximum grasp strength (51), maximal voluntary isometric torque (53), muscle strength knee flexor/extensor (54), and muscle strength toe flexor (54).

Other kinetic indexes (Table 2) were found to be less significant for characterizing the motor performance of analyzed subjects: muscle strength shoulder internal/external rotators (54), muscle strength elbow flexors/extensor (54), muscle strength hip abductors (54), muscle strength hip internal/external rotators (54), muscle strength ankle plantar flexors/dorsiflexors (54), center of pressure (CoP) velocity (57), and CoP root mean square (RMS) (57).

#### sEMG parameters

In three studies (56, 58, 60), four sEMG indexes were identified to be useful results for guiding therapy and determining the level of return to work of subjects with LBP: muscle onset (56), latency time (56), principal component score (58, 60); and EMG ensemble average waveforms (60).

The other sEMG parameter (Table 2), mean muscle activation (59), was found to be less significant for characterizing the motor performance of LBP subjects.

#### Postural parameters

In two studies, three indexes referring to posture were identified, which provide novel information concerning the effectiveness of various rehabilitation approaches for individuals with SS (55) and SCI (57), with the aim of adequate job reintegration: postural transition duration (55), aborted postural transition attempts (55), and instability index (57).

#### Other parameters

In two studies, one on HS (47) and one on SS (50), three other indexes were identified to be useful for assessing motor performance: mean of magnitude ratio of activity intensity (47), upper-limb performance (47), and reach path ratio (50), while the flexion/extension ratio evaluated in Cimarras-Otal et al. (49) was found to be less significant for characterizing the motor performance of LBP subjects (Table 2).

### Risk of bias

**Figure 2:**
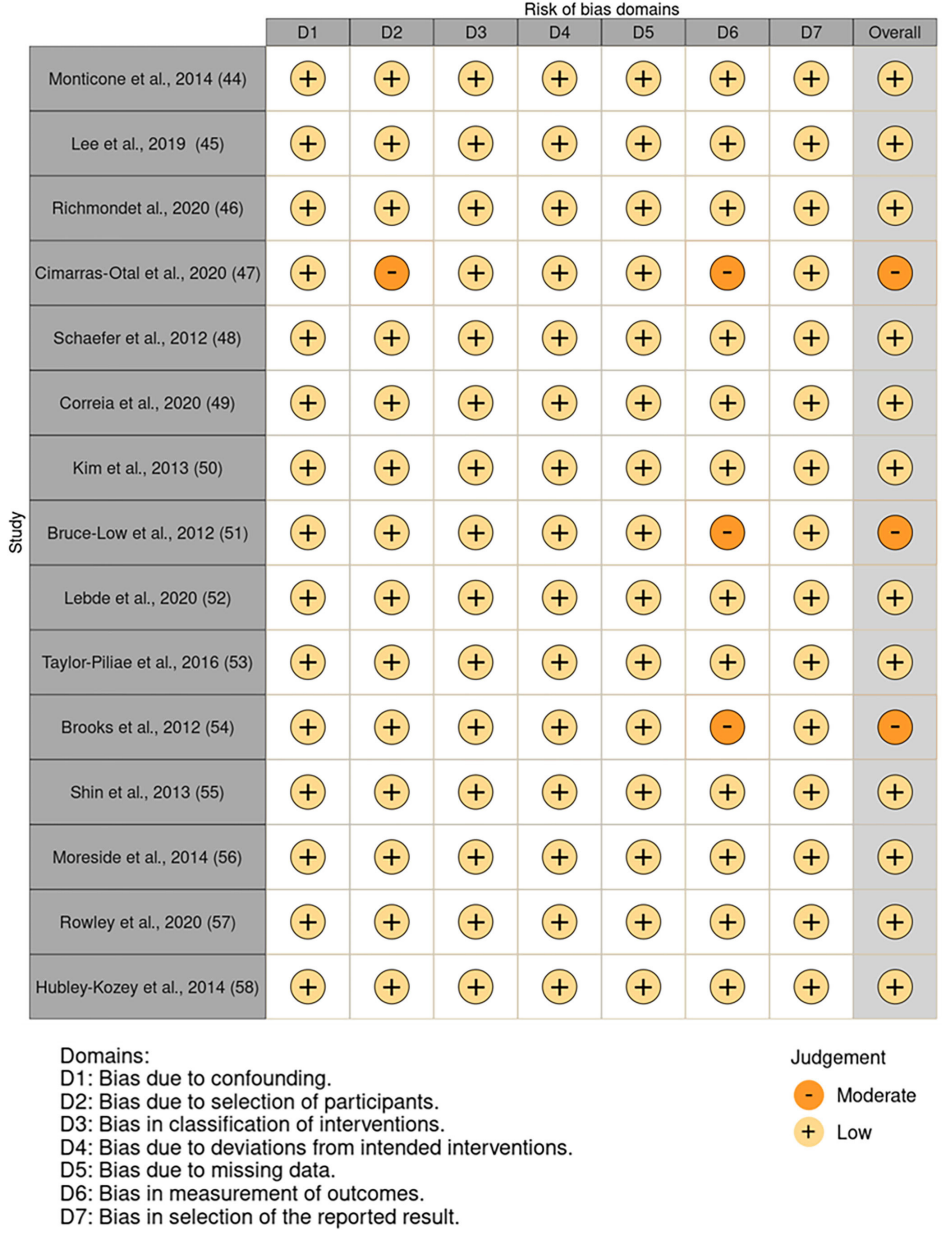
Risk of bias summary: authors' judgments for 15 included studies and for each considered domain.

**Figure 3:**
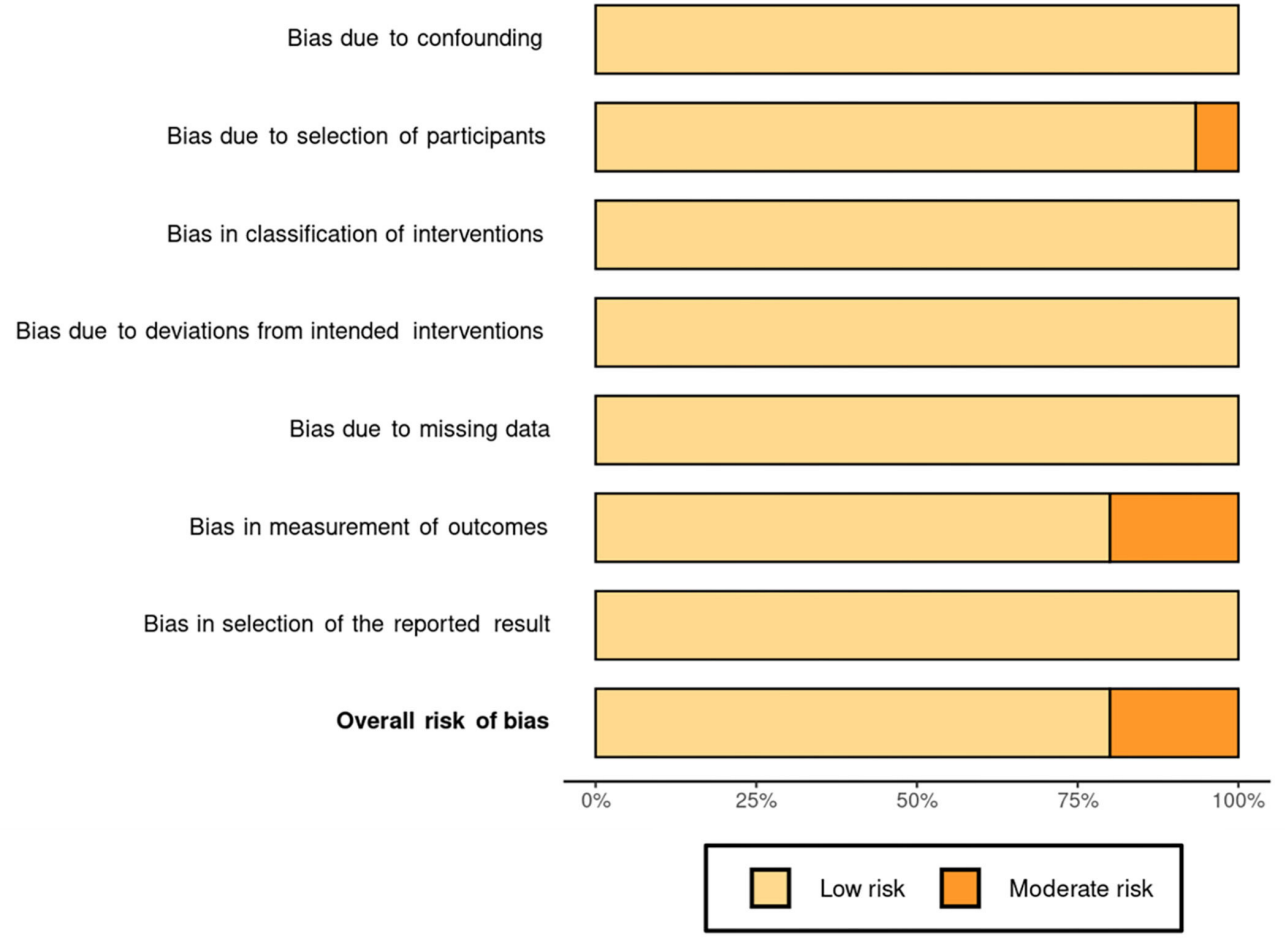
Risk of bias graph: authors' judgments for each risk of bias reported as percentages of the different studies included in the review.

The results of the risk of bias assessment are reported in the risk of bias summary (Figure 2)—where the authors' judgments are shown for all the seven considered domains and for each study included in this review, according to Higgins and Green (43), McGuinness (44), and Sterne et al. (45)—and in the risk of bias graph (Figure 3), where the authors' judgments are reported for each risk of bias as percentages across the different studies included in this review.

None of the studies considered was associated with the risk of bias due to confounding (D1), in classifications of interventions (D3), due to deviations from intended interventions (D4), due to missing data (D5), and in selection of the reported result (D7).

Instead, there was a moderate risk of bias due to participant selection (D2) in a single study (47), owing to a poor description of the subjects involved. Furthermore, due to a lack of results for some of the subjects studied, three studies (49, 53, 56) had a moderate risk of bias in outcome measurement (D6).

## Discussion

This systematic review evaluates which biomechanical and physiological indexes present in the literature are the most appropriate to evaluate the effectiveness and efficiency of workplace rehabilitation and work integration interventions for people with neuromuscular disorders that frequently face issues with employment, work difficulties, and premature work interruptions. To reach this aim, the authors found only 15 eligible articles (46–60).

The characteristics of the experimental procedures of these eligible articles suggested some interesting considerations. The samples involved in the studies were constituted by males (618) and females (612) equally, suggesting that findings related to the biomechanical and physiological indexes of motor performance can be generalized for gender. Regarding the age, all the included studies were on people of working age (18 < years <67), and we can affirm that the finding of this study can be generalized for the working age because the age range considered is wide: the subjects' mean age varied from <21.36 (57) up to 74 (55) years.

All the articles resulting from the selection process were published in the last decade, the oldest (three articles) were published in 2012 (50, 53, 56) and the most recent (five articles) in 2020 (48, 49, 51, 54, 59), and this suggests that there is a growing interest in the scientific community in this issue.

Regarding the investigated population, eight studies were on subjects with LBP (46, 49, 52, 53, 56, 58–60), three on spinal cord injury SCI (51, 52, 57), two studies on SS (50, 55), only one study on MS (48), and finally two on HS (47, 54). This result suggests that these biomechanical and physiological indexes of motor performance were used especially for job integration/reintegration of people with back pain disorders (eight studies on 15). This could be explained considering that low-back disorders are the highest incidence that disturbs workers (61–65) with economic cost (in terms of days away from work and work compensation costs) and impact on quality of life that many ergonomic interventions have been proposed during the past 3 decades to mitigate them. Indeed, there were many studies on other pathologies [i.e., Duchenne muscular dystrophy (66), Parkinson (67, 68)], and the indexes not being effectively used for a return to work. This aspect is very important because it suggests the possibility of working on the application of these indexes to facilitate the return to work of people with disabilities.

Furthermore, all the studies were performed in laboratory (46–60), and only three of them also in environments of real life (47, 52, 55). This aspect suggests that in the attempt to investigate the effectiveness of using these indexes in workplaces, measurements should be confirmed/tested in a real work environment.

There are many different work tasks that have been reproduced in the laboratory to evaluate the effectiveness of the different indexes investigated for the purpose of job insertion/re-insertion of individuals with these disorders. In fact, as can be seen in Table 1, 13 different tasks from lifting to walking through reaching and grasping and fine movements were reproduced, but each task was reproduced in one [e.g., lifting (60) and typical working activities (52)], two [e.g., daily activities (47, 55) and walking task (47, 48)], or at most three articles [e.g., balance (54, 57, 59), reaching and grasping activities (50, 51, 57)]. This reflects the wide variety of work tasks and the equal distribution among them.

In these tasks, a total of 41 different kinematic indexes were considered, and 31 of them were found to be useful for motor performance assessment (Table 2).

Furthermore, in addition to the numerous kinematic parameters, these studies investigated other parameters: five kinetic, three postural, and three other (see Tables 1, 2).

This shows the kinematic parameters for quantitative biomechanical indexes are useful and already widely used, while kinetic, postural, and surface electromyography are still underutilized in this area, despite their proven validity and necessity. In fact, when a worker with a neurological pathology and motor disorders returns to work, it is critical to conduct a thorough assessment of his/her residual motor function in order to properly construct and/or modify his/her workplace (3). The wide use of kinematics is likely to be associated with easier use even directly in the work environment, thanks to wearable technologies that are becoming increasingly popular in recent years and that are also easy to use even with user-friendly interfaces. On the other hand, kinetic and postural parameters are still very much constrained to the laboratory environment, even though sensorized shoes are beginning to become more widespread that can replace force platforms in the work environment (69). The critical issues hindering the widespread adoption of sEMG in return-to-work programs are mainly attributable to technical (both monitoring and control functions), methodological, and cultural limitations (3). Regarding monitoring issues, the most critical technical aspects are strongly associated with the sEMG technique such as electrode–skin impedance, noise, and electrode contact stability), while the methodological aspects are associated with

- problems linked to electrode location, size, configuration, and distance;
- presence of crosstalk signals (70);
- placement of sEMG electrodes for long hours;
- selection of the right sensor setup on the base of the neurological pathology and manual handling activity to be investigated;
- management of the sEMG amplitude normalization;
- definition of appropriate sEMG-related outcomes and normative data (3).

Fortunately, with the help of reliable reference materials and tutorials, the impact of these significant difficulties on the sEMG signal quality can be decreased. Particularly, the European Recommendations for Surface Electromyography (71), which needs to be updated, and the Atlas of Muscle Innervation Zones (72) are recommended as guides for the use of sEMG, together with recent tutorials and consensus articles (73–75). Users who are familiar with the contents of these publications and tutorials are aware of the current limits of the sEMG technique, which can only monitor a small number of superficial muscles. Despite this, the technical and methodological shortcomings of the sEMG approach can be addressed, and there is no justification for not using it in return-to-work rehabilitation plans. Furthermore, modern wearable sensors and electronic smart devices such as smartphones and tablets provide convenient workplace monitoring.

The existence of this educational gap is clearly attested to by the global social policies for people with disabilities (76, 77), such as the European Disability Strategy (2010–2020) and the directives 89/654/EEC, 2000/78/EC, and 2000/78/EC. Another example of this education gap is the Job Accommodation Network, which is part of the U.S. Department of Labor Office of Disability Employment Policy and provides a valuable method for including persons with neurological disorders in the workforce (78). Several educational activities should be established to enhance and use the best abilities available in rehabilitation engineering, physiotherapy, occupational therapy, and ergonomics to bridge the gap between the return-to-work idea and other disciplines. Scientific societies in the fields of physiotherapy, ergonomics, occupational medicine, biomechanics, electrophysiology, and kinesiology should promote or, at the very least, supervise and monitor bachelor, master, and PhD programs, and should include continuing education courses on the use of the management of the monitoring and HRC technologies and instrumental recordings (i.e., kinematic, kinetic, and sEMG) specifically geared to teachers in these fields (3). Furthermore, it is absolutely necessary to also include these quantitative instrumental approaches in the international ergonomic standards (79–82).

The bias affecting these results is low overall (Figure 2), so the results can be considered reliable. In fact, only three articles of 15 showed moderate bias (49, 53, 56), and two articles (53, 56) of these three are quite old, both published in 2012.

The examined articles do not refer to the experts who conducted the analyses or who will be required to do so in future, except in four articles where they mentioned physiotherapists, physiatrists, and psychologists (46, 53, 56, 60). We believed that the professionals trained for innovative job accommodation programs, however, should be occupational health and safety technicians and ergonomists who work in a multidisciplinary team also made up of neurologists, occupational physicians, physiatrists, physiotherapists, biomedical engineers, and movement scientists.

In summary, the results of this review show that quantitative biomechanical and physiological indexes are good tools to be used for job integration/reintegration and widely used the kinematic ones, and the electromyographic ones still little used. Indeed, by contributing to prevent the loss of employment and improve overall quality of life, the use of appropriate indexes for motor monitoring in job integration programs of people with neuromuscular diseases will allow to assess the effectiveness of the ergonomic interventions (1, 3). In addition, their utilization will enable more comprehensive job accommodation plans that can incorporate workplace and work task rehabilitation (35–37). The Industry 4.0 era allows for the deployment of human–robot collaboration (HRC) technologies that may support people in real time at their workplaces based on their motor requirements. In this setting, wearable miniaturized sensors, kinematic, kinetic, and sEMG-based indexes can be used to control collaborative robots; classify residual motor functions; and assess pre–post-rehabilitation and ergonomic therapies.

### Limitation of the present systematic review

The main limitations of this systematic review are related to the small number of articles that resulted in being eligible, which was mainly due to the narrow eligibility criteria oriented to the occupational field and the quantitative biomechanical indexes. The number of articles grows significantly if we also consider studies based on qualitative (i.e., studies with only questionnaires) and not quantitative indexes and if we do not limit the application to the work environment. Furthermore, another limitation is that the systematic review was not registered in PROSPERO (https://www.crd.york.ac.uk/prospero/).

## Data availability statement

The original contributions presented in the study are included in the article/supplementary materials, further inquiries can be directed to the corresponding author.

## Author contributions

GC, LF, AT, TV, FD, and AR planned the manuscript, performed the literature searches, wrote the text, and revised the manuscript. All authors contributed to the article and approved the submitted version.

## Funding

The research presented in this article was carried out as part of the SOPHIA Project, which has received funding from the European Union's Horizon 2020 Research and Innovation Program under Grant Agreement No. 871237.

## Conflict of interest

The authors declare that the research was conducted in the absence of any commercial or financial relationships that could be construed as a potential conflict of interest.

## Publisher's note

All claims expressed in this article are solely those of the authors and do not necessarily represent those of their affiliated organizations, or those of the publisher, the editors and the reviewers. Any product that may be evaluated in this article, or claim that may be made by its manufacturer, is not guaranteed or endorsed by the publisher.
